# Supplementary material for: Dual functions of SPOP and ERG dictate androgen therapy responses in prostate cancer
Source: Nat Commun. 2021 Feb 2;12:734. doi: 10.1038/s41467-020-20820-x (PMC7854732; doi:10.1038/s41467-020-20820-x)
Supplement: Supplementary file 3 — Description of Additional Supplementary Files [file 41467_2020_20820_MOESM3_ESM.pdf]

Description of Additional Supplementary Files

Supplementary Data 1. "Gene signatures characteristic of SPOP-mutations and ERG-fusions identified in primary tumors (TCGA) and applied to CRPC (SU2C) and PDX models. SPOP-specific gene-signatures were determined as discussed in Materials and Methods."

Supplementary Data 2. Differential AR binding sites between SPOP-mutant and ERG-rearranged tumors (GSE28950) as determined by DiffBind and DESeq2. Shown are peaks with an FDR adjusted p-value <0.05. ChIPSeeker was used to annotate peaks.

Supplementary Data 3. The genes belonging to the custom AR and ERG-specific gene signatures are derived as determined in Materials and Methods.

Supplementary Data 4: "Averaged gene-expression values (vst-transformed) for Control, wild-type, and mutant SPOPoverexpressing VCaP cells. Provided are differential gene-expression statistics (DESeq2) for the three comparisons; SPOP-WT vs. Control, SPOP-Mut vs. Control, and SPOP-Mut vs. SPOP-WT. Provided are annotations respective to the belonging of individual genes to the identified AR and ERG specific gene-signatures."

Supplementary Data 5. " AR and ERG specific Enrichment analysis gene-sets determined comparing SPOP-Mutant vs. SPOP-wildtype VCaP cells. Enrichment was determined using Camera pre-ranked (limma package) as described in Material and Methods. "

Supplementary Data 6. "Enrichment analysis of HALLMARK gene-sets (MSigDB) determined for the comparisons: SPOP-Mutant vs. SPOP-Wildtype, SPOP-Mutant vs. Control, SPOP-Wildtype vs. Control VCaP cells. Enrichment was determined using Camera pre-ranked (limma package) as described in Material and Methods. MYC_TARGETS_V1 and MYC_TARGETS_V2 were fused into a single gene list."

Supplementary Data 7. "Enrichment analysis of AR and ERG specific gene-sets determined for the comparison between SPOP-Mutant overexpressing VCaP cells vs. Control. Enrichment was determined using Camera pre-ranked (limma package) as described in Material and Methods. "

Supplementary Data 8. This Supplementary Data contains averaged gene-expression values (vst-transformed) for Control, wild-type, and mutant SPOP-overexpressing LNCaP cells in the

presence/absence of concomitant DERG overexpression. Provided are differential gene-expression statistics (DESeq2) to compare SPOP-Mutant vs. SPOP-WT overexpressing LNCaP cells in ERG wild type and DERG setting.

Supplementary Data 9. "Enrichment analysis of HALLMARK gene-sets (MSigDB) determined for the comparisons: SPOP-Mutant vs. SPOP-Wildtype overexpression LNCaP cells, in the presence or absence of DERG. Enrichment was determined using Camera pre-ranked (limma package) as described in Material and Methods. MYC_TARGETS_V1 and MYC_TARGETS_V2 were fused into a single gene list."

Supplementary Data 10. Normalized ssGSEA scores were computed for AR and ERG specific genesets in primary tumors from the TCGA cohort. Scores are averaged by tumor-subtype and rescaled by row. Patients were assigned into subgroups (SPOP/ERG/OTHER) as described in materials and methods. Samples, including other ETSrearrangements, were excluded. Subgroup OTHER was used as a reference.

Supplementary Data 11. Reported are ssGSEA scores determined for the custom AR and ERG specific gene-signatures and computed for each patient of the TCGA cohort. Indicated is the tumor subtype the patient was associated to as described in Materials and Methods.

Supplementary Data 12. Averaged gene-expression values (vst-transformed) of Primary Prostate Tumors derived from the TCGA dataset. Samples are grouped into 3 subtypes (SPOP/ERG/OTHER) as described in Materials and Methods. Provided are differential gene-expression statistics (DESeq2) for the comparison between SPOP-Mutant samples and ERG-rearranged tumors. Provided are annotations respective to the belonging of individual patients to the respective tumor subtype.

Supplementary Data 13. Enrichment analysis of AR and ERG specific gene-sets determined for the comparison between SPOP-Mutant and ERG-rearranged primary tumors from the TCGA cohort. Enrichment was determined using Camera pre-ranked (limma package) as described in Material and Methods.

Supplementary Data 14. Proteome data, detected using the tandem mass tag (TMT)-based quantitative massspectrometry, in VCaP cancer cell line expressing Control, SPOP-WT, SPOP-Y87C, SPOP-F102C, SPOP-W131G, sample are in duplicate.

Supplementary Data 15. Proteome data matched with the RNA-seq data in VCaP Cancer cell line overexpressing SPOPWT, SPOP-Y87C, SPOP-F102C, SPOP-W131G. Highlighted in RED are Gene, whose protein expression was upregulated without concomitant RNA expression.

Supplementary Data 16. "Averaged gene-expression values (vst-transformed) for PLKO and ZMYND11-KO VCaP cells using two different hairpins. Provided are differential gene-expression statistics (DESeq2) for comparison: ZMYND11- KO vs. PLKO. Averaged expression of individual hairpins and PLKO is also included. Provided are annotations respective to the belonging of individual genes to the identified AR and ERG specific gene-signatures."

Supplementary Data 17. "Averaged gene-expression values (vst-transformed) for Control, HA-ZMYND11-DM/BD, and HA-ZMYND11-DM VCaP cells. Provided are differential gene-expression statistics (DESeq2) for comparison: HAZMYND11-DM vs. Control. Provided are annotations respective to the belonging of individual genes to the identified AR and ERG specific gene-signatures."

Supplementary Data 18. "Enrichment analysis of AR and ERG specific gene-sets determined for the comparison between HA-ZMYND11-DM overexpressing VCaP cells vs. Control. Enrichment was determined using Camera preranked (limma package) as described in Material and Methods. "

Supplementary Data 19. List of ZMYND11 peaks identified from ChIP-Seq experiment performed in Control VCaP cells. Peaks were called using Macs2 as detailed in Materials and Methods. Annotation of peaks was performed using ChIPseeker.

Supplementary Data 20. List of ZMYND11 peaks identified from ChIP-Seq experiment performed in wildtype-SPOP overexpressing VCaP cells. Peaks were called using Macs2 as detailed in Materials and Methods. Annotation of peaks was performed using ChIPseeker.

Supplementary Data 21. List of ZMYND11 peaks identified from ChIP-Seq experiment performed in mutantSPOP(Y87C) overexpressing VCaP cells. Peaks were called using Macs2 as detailed in Materials and Methods. Annotation of peaks was performed using ChIPseeker.

Supplementary Data 22. Enrichment of ZMYND11 peaks identified from ChIP-Seq experiments performed in SPOPMutant (Y87C) overexpressing VCaP-cells. Reported are Statistics determined for AR and ERG-specific gene-signatures that result positively enriched. The analysis was performed using ChIPenrich as described in Materials and Methods.

Supplementary Data 23. "Averaged gene-expression values (vst-transformed) for PLKO, and SPOP-KO VCaP cells using two different hairpins. Provided are differential gene-expression statistics (DESeq2) for comparison: SPOP-KO vs PLKO. Averaged expression of individual hairpins and PLKO is also included. Provided are annotations respective to the belonging of individual genes to the identified AR and ERG specific gene-signatures."

Supplementary Data 24. "Enrichment analysis of AR and ERG specific gene-sets determined for the comparison between SPOP-KO and Control VCaP cells. Enrichment was determined using Camera pre-ranked (limma package) as described in Material and Methods."

Supplementary Data 25. "Averaged gene-expression values (vst-transformed) for DHT treated (100nM) and ,Control VCaP cells.. Provided are differential gene-expression statistics (DESeq2) for comparison: DHT vs CTRL. Provided are annotations respective to the belonging of individual genes to the identified AR and ERG specific gene-signatures."

Supplementary Data 26. "Enrichment analysis of AR and ERG specific gene-sets determined for the comparison between DHT treated (100nM) and Control VCaP cells. Enrichment was determined using Camera pre-ranked (limma package) as described in Material and Methods."
