# Supplementary material for: Dual functions of SPOP and ERG dictate androgen therapy responses in prostate cancer
Source: Nat Commun. 2021 Feb 2;12:734. doi: 10.1038/s41467-020-20820-x (PMC7854732; doi:10.1038/s41467-020-20820-x)
Supplement: Supplementary file 30 — Reporting Summary [file 41467_2020_20820_MOESM30_ESM.pdf]

nature research

Corresponding author(s): Prof. Jean-Philippe Theurillat

Last updated by author(s): Oct 20, 2020

Reporting Summary

Nature Research wishes to improve the reproducibility of the work that we publish. This form provides structure for consistency and transparency in reporting. For further information on Nature Research policies, seeAuthors & Referees and theEditorial Policy Checklist .

Statistics

For all statistical analyses, confirm that the following items are present in the figure legend, table legend, main text, or Methods section.

n/a	Confirmed	
☐	☒	The exact sample size (*n*) for each experimental group/condition, given as a discrete number and unit of measurement
☐	☒	A statement on whether measurements were taken from distinct samples or whether the same sample was measured repeatedly
☐	☒	The statistical test(s) used AND whether they are one- or two-sided<br>*Only common tests should be described solely by name; describe more complex techniques in the Methods section.*
☐	☒	A description of all covariates tested
☐	☒	A description of any assumptions or corrections, such as tests of normality and adjustment for multiple comparisons
☐	☒	A full description of the statistical parameters including central tendency (e.g. means) or other basic estimates (e.g. regression coefficient) AND variation (e.g. standard deviation) or associated estimates of uncertainty (e.g. confidence intervals)
☐	☒	For null hypothesis testing, the test statistic (e.g. *F*, *t*, *r*) with confidence intervals, effect sizes, degrees of freedom and *P* value noted<br>*Give P values as exact values whenever suitable.*
☒	☐	For Bayesian analysis, information on the choice of priors and Markov chain Monte Carlo settings
☒	☐	For hierarchical and complex designs, identification of the appropriate level for tests and full reporting of outcomes
☐	☒	Estimates of effect sizes (e.g. Cohen's *d*, Pearson's *r*), indicating how they were calculated

Our web collection on statistics for biologists contains articles on many of the points above.

Software and code

Policy information about availability of computer code

Data collection	Gene-sets were obtained from the Hallmark collection, which is curated by the Molecular Signature DataBase (MSigDB).
Data analysis	Sequence alignments (RNA-Seq) to the reference human genome (GRCh38) was performed using STAR41 (v.2.5.2a). Gene-expression was quantified at gene level by using the comprehensive annotations made available by Gencode42. Specifically, we used v27 release of the Gene Transfer File (GTF). Raw-counts were further processed in the R Statistical environment (release v3.4.1) and downstream differential expression analysis was performed using DESeq (v1.1.16.1) pipeline. Gene-set enrichment analysis was performed using CAMERA (1.32.0) in R statistical enviroment. FastQC (v0.11.9), RSeQC (v2.6.4), AfterQC (v0.97) and Qualimap (v2.0) were used to evaluate overall sequencing quality. Reads were deduplicated priopr peak calling procedure.<br>ChIP-Seq derived reads were aligned to hg38 release of the human reference genome using bwa-mem47 (0.7.15). MACS (v.2.1.0) was used to perform peak calling procedure using a cutoff FDR q-value of 0.01 and a mappable genome size optimized for hg38 equal to 2.9 gigabases. Intersection of peaks was berformed using bedtools (v2.28.0). Downstream analysis was performed in R statistical environment. ChIPseeker (v1.12.1) was used to annotate peaks.<br>Protei-protein interaction networks were generated using Cytoscape (v3.7.2).

For manuscripts utilizing custom algorithms or software that are central to the research but not yet described in published literature, software must be made available to editors/reviewers. We strongly encourage code deposition in a community repository (e.g. GitHub). See the Nature Research guidelines for submitting code & software for further information.

Data

Policy information about availability of data

All manuscripts must include a data availability statement. This statement should provide the following information, where applicable:

- Accession codes, unique identifiers, or web links for publicly available datasets
- A list of figures that have associated raw data
- A description of any restrictions on data availability

RNA-seq and ChIP-Seq data generated were available and have been deposited in the ArrayExpress database at EMBL-EBI and were assigned the accessions E-MTAB-7165, E-MTAB-7170, E-MTAB-7173, E-MTAB-7174 (ChIP-Seq).
Samples SRR3713255, SRR3713256,SRR3713257, SRR3713267, SRR3713268, SRR3713269, SRR3713270, SRR3713271, SRR3713272 were retrieved from SRA (Short-Read-Archive) using fastq-dump, which is part of SRA-toolkit managed by NCBI. Publicly available RNA-Seq data for primary prostate cancer were obtained from The Caner Genome Atlas (TCGA) database and retrieved from Genomics Data Commons (GDC) in form of gene-centric raw counts, computed using HTSeq. Information respective to public data (TCGA and other datasets) are included into the 'data' section.

Field-specific reporting

Please select the one below that is the best fit for your research. If you are not sure, read the appropriate sections before making your selection.

[x] Life sciences [ ] Behavioural & social sciences [ ] Ecological, evolutionary & environmental sciences

For a reference copy of the document with all sections, see nature.com/documents/nr-reporting-summary-flat.pdf

Life sciences study design

All studies must disclose on these points even when the disclosure is negative.

Sample size	No sample size calculation was performed for this study. In order to perform statistic analyses and achieve statistic significances, we mostly chose sample sizes of 3-6 for our in vitro experiments, and 4-12 for our in vivo experiments.
Data exclusions	In order to recapitulate the levels of supraphysiological testosterone administrated in clinical trials (explain in the methods section), mice reaching at least 3 times the testosterone levels measured before the treatment initiated were included in the depicted data. Mice that died from unexpected illness along the experiment were excluded, as the measurement of tumor size could not continue.
Replication	All attempts at replications were successful. For each experiments, the number of biological independent experiments is reported in the figure legends
Randomization	All samples/animals were randomly allocated to experimental groups and processed.
Blinding	Experiments were not blinded. However, we followed standard laboratory procedures of randomization. Each experiment was designed with proper controls, and samples for comparison were collected and analyzed under the same conditions.

Reporting for specific materials, systems and methods

We require information from authors about some types of materials, experimental systems and methods used in many studies. Here, indicate whether each material, system or method listed is relevant to your study. If you are not sure if a list item applies to your research, read the appropriate section before selecting a response.

Materials & experimental systems

n/a	Involved in the study	
[ ]	[x]	Antibodies
[ ]	[x]	Eukaryotic cell lines
[x]	[ ]	Palaeontology
[ ]	[x]	Animals and other organisms
[x]	[ ]	Human research participants
[x]	[ ]	Clinical data

Methods

n/a	Involved in the study	
[ ]	[x]	ChIP-seq
[x]	[ ]	Flow cytometry
[x]	[ ]	MRI-based neuroimaging

Antibodies

Antibodies used	anti-SPOP (ab81163, Abcam)

anti-TRIM24 (Sc-271266, Santa Cruz)
anti-ß-ACTIN (4967, Cell Signaling)
anti-AR (Sc-7305, Santa Cruz)
anti-GADPH (Sc-47724, Santa Cruz)
anti-ERG (Sc-271048, Santa Cruz)
anti-VCL (SAB1404522, Sigma)
anti-ZMYND11(NBP2-20960, Novus Biologicals)
anti-HA (H3663, Sigma)
anti-BRD2 (A302-583A, Bethyl Labs)
anti-NCOA3 (2126, Cell Signaling)
anti-DEK (610948, BDBioscience)
anti-p21 (2947S, Cell Signaling)
anti-c-MYC (5605S, Cell Signaling)
anti-HOXB13 (Sc-28333, Santa Cruz)
anti-PTEN (9559, cell signaling)
anti-p21 (ab188224, Abcam)
anti-HOXB13 (NBP2-43655, Novus biologicals)
anti-p16 (ab211542, Abcam, 1:1200)
anti-Ki67 (Clone SP6; Lab Vision Corporation #RT-9106-R7, RTU)
anti-Phospho-HP1y (Ser83) Antibody (CST #2600, 1:200)
anti-CK8 (ab,59400, Abcam)
anti-CK5 (ab52635, Abcam)
anti-GDF-15 (27455-1-AP, proteintech)
horseradish peroxidase-conjugated secondary antibody, (W4028 or W4018 Promega)

Validation

Antibody validated in this and previous study:
anti-SPOP (ab81163, Abcam), anti-TRIM24 (Sc-271266, Santa Cruz), anti-ERG (Sc-271048, Santa Cruz), anti-ZMYND11 (NBP2-20960, Novus Biologicals), anti-HA (H3663, Sigma), anti-AR (Sc-7305, Santa Cruz), the followed antibody were further validated in this study using Knock-down or overexpression of the target protein assessed by immunoblotting.

anti-BRD2 (A302-583A, Bethyl Labs) was validated in our previous study: https://pubmed.ncbi.nlm.nih.gov/28805821/

ABCAM antibodies validation steps:
Abcam validation : https://www.abcam.com/primary-antibodies/how-we-validate-our-antibodies

Antibodies are validated in western blot using lysates from cells or tissues that we have identified to express the protein of interest. Once we have determined the right lysates to use, western blots are run and the band size is checked for the expected molecular weight. We will always run several controls in the same western blot experiment, including positive lysate and negative lysate.

When possible, we also include knock-out (KO) cell lines as a true negative control for our western blots. We are always increasing the number of KO-validated antibodies we provide. In addition, we run old stock alongside our new stock. If we know the old stock works well, this also acts as a suitable positive control.
Immunohistochemistry and immunocytochemistry
IHC and ICC determine whether an antibody recognizes the correct protein based on cellular and subcellular localization. Antibody specificity is confirmed by looking at cells that either do or do not express the target protein within the same tissue. Initially, our scientists will review the available literature to determine the best cell lines and tissues to use for validation. We then check the protein expression by IHC/ICC to see if it has the expected cellular localization (Figure 3). If the localization of the signal is as expected, this antibody will pass and is considered suitable for use in IHC/ICC.
We use a variety of methods, including staining multi-normal human tissue microarrays (TMAs), multi-tumor human TMAs, and rat or mouse TMAs during antibody development. These high-throughput arrays allow us to check many tissues simultaneously, providing uniformly as all tissues are exposed to the exact same conditions.

Cell signaling antibodies validation steps:
If the western blot result gives a clear, clean band, and we are happy with the result from the control lanes, these antibodies will be passed and added to the catalog Cell signaling validation policies:
https://www.cellsignal.com/contents/our-approach/cst-antibody-validation-principles/ourapproach-validation-principles

Proteintech antibodies validation steps:
Source: https://www.lubio.ch/supplier/proteintech/#:~:text=Proteintech%20are%20currently%20developing%20their,still%20an%20important%20target%20today.

Novusbio anti-HOXB13 :
Here the validation blot for HOXB13 in PC3 prostate cancer cells:
https://www.novusbio.com/products/hoxb13-antibody_nbp2-43655

anti-DEK (610948, BDBioscience) :
Antibody has been used previous in the following research articles:
https://pubmed.ncbi.nlm.nih.gov/28805821/
https://pubmed.ncbi.nlm.nih.gov/25278611/

anti-HOXB13 (Sc-28333, Santa Cruz)
Relevant publications using the same antibody
https://pubmed.ncbi.nlm.nih.gov/32499640/
https://pubmed.ncbi.nlm.nih.gov/30560549/
https://pubmed.ncbi.nlm.nih.gov/30661984/

anti-GADPH (Sc-47724, Santa Cruz)
Antibody's webpage:
https://www.scbt.com/p/gapdh-antibody-0411?requestFrom=search
Anti-GAPDH Antibody (0411) is a mouse monoclonal IgG1 κ GAPDH antibody, cited in 1,896 publications.
Relevant publications:
https://pubmed.ncbi.nlm.nih.gov/31320625/
https://pubmed.ncbi.nlm.nih.gov/31320625/

anti-VCL (SAB1404522, Sigma)
Antibody's webpage:
https://www.sigmaaldrich.com/catalog/product/sigma/sab1404522?lang=fr&region=CH
https://pubmed.ncbi.nlm.nih.gov/25183785/
https://pubmed.ncbi.nlm.nih.gov/25893857/

anti-Ki67 (Clone SP6; Lab Vision Corporation #RT-9106-R7, RTU
Please find below a list of relevant publication for the antibody anti-Ki67
https://www.labome.com/product/Invitrogen/MA5-14520.html

Eukaryotic cell lines

Policy information about cell lines

Cell line source(s)	VCaP, LNCaP, PC3, 22Rv1, HEK293 cells were purchased from ATCC. LAPC-4 were a gift from Prof. Helmut Klocker from the Universitätsklinik für Urologie - Labor Anichstraße 35, 6020 Innsbruck (originally from The Charles Sawyers Lab).
Authentication	The cell lines were purchased and autenticated by ATCC.
Mycoplasma contamination	All cell lines were regularly tested and were negative for mycoplasma (MycoAlert Mycoplasma Detection kit).
Commonly misidentified lines (See ICLAC register)	n/a

Animals and other organisms

Policy information about studies involving animals; ARRIVE guidelines recommended for reporting animal research

Laboratory animals	All animal experiments were carried out in male athymic nude mice (Balb/c nu/nu, 6-8 weeks old), NSG mice (NOD Scid Gamma, 6-8 weeks old), and NRG (NOD Rag gamma, 6-8 weeks old), housing condition are in the methods section of the manuscript.
Wild animals	The study did not involve wild animals.
Field-collected samples	The study did not involve field-collected samples.
Ethics oversight	All animal experiments were carried out accordingly to protocol approved by the Swiss Veterinary Authority (No. TI-14-2014, TI-38-2018, TI-39-2018 and TI-42-2018).

Note that full information on the approval of the study protocol must also be provided in the manuscript.

ChIP-seq

Data deposition

- [x] Confirm that both raw and final processed data have been deposited in a public database such as GEO.
- [x] Confirm that you have deposited or provided access to graph files (e.g. BED files) for the called peaks.

Data access links<br>*May remain private before publication.*	https://www.ebi.ac.uk/arrayexpress/help/how_to_search_private_data.html<br>Username: Reviewer_E-MTAB-7174<br>Password: onwwwwni
Files in database submission	VCAP_CTRL_INPUT.fastq.gz<br>VCAP_CTRL.fastq.gz<br>VCAP_WT_INPUT.fastq.gz<br>VCAP_WT.fastq.gz<br>VCAP_Y87C_INPUT.fastq.gz<br>VCAP_Y87C.fastq.gz<br>VCAP_CTRL.narrowPeak (bed file)<br>VCAP_WT.narrowPeak (bed file)<br>VCAP_Y87C.narrowPeak (bed file)
Genome browser session<br>(e.g. UCSC)	UCSC Genome broser shared session:<br>https://genome-euro.ucsc.edu/cgi-bin/hgTracks?<br>hgS_doOtherUser=submit&hgS_otherUserName=marcobolis&hgS_otherUserSessionName=ZMYND11_VCAP<br>(otherwise access is granted using username marcobolis, session name ZMYND11_VCAP)

Methodology

Replicates	1
Sequencing depth	75bp-long single-end reads were sequenced using Illumina NextSeq500.<br>VCAP_CTRL_INPUT.fastq (47 Million reads)<br>VCAP_CTRL.fastq (19 Million reads)<br>VCAP_WT_INPUT.fastq (48 Million reads)<br>VCAP_WT (51 Million reads)<br>VCAP_Y87C_INPUT (42 Million reads)<br>VCAP_Y87C (41 Million reads)
Antibodies	anti-ZMYND11 antibody (NBP2-20960, Novus Biologicals)
Peak calling parameters	- peaks were called on VCAP_CTRL.fastq using its matched input file: VCAP_CTRL_INPUT.fastq<br>- peaks were called on VCAP_WT.fastq using its matched input file: VCAP_WT_INPUT.fastq<br>- peaks were called on VCAP_Y87C.fastq using its matched input file: VCAP_Y87C_INPUT.fastq<br>FDR threshold used for peak calling (using MACS v2.1.0) was set to 0.01 (-q 0.01) . The mappable genome size was set to 2.9 gigabases (-g 2.9e9)
Data quality	FastQC (v0.11.9), RSeQC (v2.6.4), AfterQC (v0.97) and Qualimap (v2.0) were used to evaluate overall sequencing quality.<br>Reads were deduplicated priopr peak calling procedure.<br>In our manuscript we used FDR < 0.01. The numbers of peaks showing an enrichment above 5-fold are 5399 out of 7646 total peaks identified in VCAP_Y87C cells and 1673 out of 2072 total peaks identified in VCAP_CTRL.
Software	Reads were aligned to hg38 release of the human reference genome using bwa-mem (0.7.15). MACS (v.2.1.0) was used to perform peak calling procedure using a cutoff FDR q-value of 0.01 and a mappable genome size optimized for hg38 equal to 2.9 gigabases. Downstream analysis was performed in R statistical environment. Intersection of peaks was berformed using bedtools (v2.28.0). ChIPseeker(v1.12.1) was used to annotate peaks and to represent the distribution of ZMYND11 binding sites relative to Transcription Start Sites (TSSs).
